# Brazilian dental teleconsulting: dentist satisfaction and associated factors

**DOI:** 10.1590/1807-3107bor-2025.vol39.027

**Published:** 2025-05-23

**Authors:** Lígia Cristelli PAIXÃO, Mauro Henrique Nogueira Guimarães ABREU, Antônio Paulino RIBEIRO-SOBRINHO, Renata Castro MARTINS

**Affiliations:** (a) Universidade Federal de Minas Gerais - UFMG, School of Dentistry, Belo Horizonte, MG, Brazil.; (b) Universidade Federal de Minas Gerais - UFMG, School of Dentistry, Department of Community and Preventive Dentistry, MG, Brazil.; (c) Universidade Federal de Minas Gerais - UFMG, School of Dentistry, Department of Restorative Dentistry, Belo Horizonte, MG, Brazil.

**Keywords:** Telemedicine, Primary Health Care, Dentistry, COVID-19

## Abstract

Teleconsulting consists of communication between professionals about clinical matters using information and communication technologies. Satisfaction is an important outcome in the evaluation of telehealth programs and an indicator of the effectiveness of the services offered. This cross-sectional study evaluated dentist satisfaction with asynchronous dental teleconsulting services offered by the Telehealth Brazil Networks Program and associated factors during 2020, using a secondary database: the Monitoring and Evaluation System of Telehealth Results. The outcome was the satisfaction of the dentist with the response of the teleconsulting session. The other variables collected were the dentist’s sex and specialty, the time at which the question and answer of the teleconsulting session were submitted, patient referral, and the dental specialty addressed in the teleconsulting session. The variables were analyzed descriptively using frequency, and binary logistic regression models were used to measure the association between satisfaction and factors related to the dentist and the teleconsulting service. A total of 1,719 dental teleconsulting sessions were conducted. A 26.7% satisfaction level was achieved. Satisfaction with teleconsulting was indicated by the association between professionals declaring that dental teleconsulting “avoided referrals” (OR =1.55, 95%CI: 1.17–2.04, p = 0.002) and the teleconsulting sessions in the field of oral medicine (OR = 0.61, 95%CI: 0.43–0.87, p = 0.006). Dentists’ satisfaction with asynchronous dental teleconsulting was low. Dentists reporting that teleconsulting avoided patient referrals to other care levels were more likely to be satisfied with the answer from the teleconsultant. Moreover, dentists who submitted oral medicine-related questions were significantly less satisfied with the teleconsulting feedback than those who submitted questions regarding other fields.

## Introduction

Telehealth is the use of information and communication technology (ICT) for remote healthcare^1-3,^ and provides interactive access to expert opinion to treat patients.^2,3^ It has also enabled early diagnosis,^4^ helped address the disparate distribution of specialized healthcare services^2^ by supporting remote multidisciplinary care,^1,3,4^ and promoted continuing education for health professionals.^3,5^ Telehealth has ensured excellent health service outcomes, and is broadly used, including teleconsultations, web conferences, tele-education, telediagnosis, teletriage, and telemonitoring.^2-4^ Since 2020, the COVID-19 pandemic has highlighted certain attributes of telehealth, especially because it enabled certain healthcare services to continue while ensuring social distancing.^6^ Telehealth also avoids unnecessary displacements, and helps reduce referral waiting time, saving financial resources both for the health system and patients. In dentistry, this remote healthcare is referred to as teledentistry.^3^

Brazil is characterized by socioeconomic contrasts, different levels of professional preparation, and unequal access to healthcare.^7^ The Ministry of Health implemented the Telehealth Brazil Networks Program to strengthen, address, and resolve both primary health care (PHC) issues and access to specialized health in Brazil.^5,7^ As of 2006, the program was expanded and redefined to include the entire country.^5,7^

Teleconsulting is one of the program’s strategies and consists of communications between the PHC professional and a teleconsultant (expert in a specific field). The messages address clinical matters, health promotion actions, and dental practice (work processes). Telehealth centers offer teleconsulting services that are communicated synchronously by messaging and videoconferencing or asynchronously by dialogues that must be answered within 72 hours.^3,5^ Initially, Brazilian regulations stated that remote dentistry was allowed only among health professionals.^1^ Nevertheless, the COVID-19 pandemic required the approval of Resolution #226/2020 by the Federal Dentistry Council, which allowed telemonitoring (remote monitoring of patients undergoing treatment by dentists) and teletriage (applying a pre-clinical questionnaire to define the best time to treat these patients).^8^ Therefore, telehealth services started to be used more frequently by health professionals^9^. Since 2022, the remote provision of services related to all health professions has been permitted in the country.^10^

Satisfaction is an important outcome in the evaluation of telehealth programs, indicating the effectiveness of the services offered.^11,12^ Professional satisfaction is a good predictor of the use of technology, adhesion to treatment, and correct use of healthcare services by patients.^12,13^

Considering the risks and barriers to optimal health outcomes in Brazil,^7^ the continuous evaluation of the program is required to monitor the quality of care being offered, identify gaps, and improve its effectiveness. Hence, this study aimed to evaluate dentists’ satisfaction and associated factors in the asynchronous dental teleconsulting of the program in 2020. The null hypothesis was that there is no association between dentist satisfaction and factors related to dentists and dental teleconsulting.

## Methods

The Research Ethics Committee of our Institution approved the study under the code CAAE 17400319.9.0000.5149. This cross-sectional analytical study assessed a secondary database of all asynchronous dental teleconsulting of the Telehealth Brazil Networks Program in 2020, during the COVID-19 pandemic. Data were collected from the Monitoring and Evaluation System of Telehealth Results (SMART-Sistema de Monitoramento e Avaliação dos Resultados do Telessaúde, in Portuguese), which integrates information on telehealth centers into the program.^14^ The access to the question and answer from each teleconsulting session was not available at the moment of data extraction from SMART. Teleconsulting data were then filtered according to codes from the International Classification of Diseases (ICD-10) and the International Classification of Primary Care 2 (ICPC-2) applied to dentistry.^15^ Although dental teleconsulting sessions may be requested by other health professionals, only those requested by dentists were extracted from SMART and analyzed in this study.

The study outcome was the satisfaction of the PHC dentist with the teleconsulting session answer. The variable had five categories based on the dentist’s feedback given at the end of the teleconsulting: very satisfied, satisfied, neither satisfied nor dissatisfied, dissatisfied, and very dissatisfied, later dichotomized into very satisfied/satisfied and dissatisfied/neither satisfied nor dissatisfied/very dissatisfied. The decision to use a dichotomous variable combining the categories “neither satisfied nor dissatisfied” and “dissatisfied/very dissatisfied” was because they all failed to express the satisfaction of the professional with the teleconsulting session.

Covariables were defined according to the data available in SMART, and included both the dentist-related factors of sex (female/male) and dentist specialty (Family Health Strategy (FHS)/general dental practitioner/specialist) and the teleconsulting-related factors time of questions and time of answers (during working hours or not) and prevention of patient referral (yes/no). “Family Health Strategy” was considered a public health specialist, “General Dental Practitioner” was considered a non-specialist, and “Specialist” was considered a specialist in a field of dentistry other than public health. Dental fields were classified according to ICD-10 or ICPC-2: clinical dentistry (dentistry, endodontics, and periodontics); health promotion and prevention (oral health education and prophylactic measures); pharmacology (prescription and adverse effects of medications); semiology (diagnosis of general and systemic diseases such as diabetes and hypertension, without indicating oral lesions); oral medicine (oral alterations or lesions); and services (patient referral, health system and service operation, and administrative processes).

The variables were analyzed descriptively using frequency. Binary logistic regression models were used to estimate odds ratios (OR), 95% confidence intervals (95%CI), and p-values of the association between satisfaction and related factors. All variables with a p-value < 0.25 in the bivariate analysis were included in the multiple logistic regression model. Variables with a p-value <0.05 were retained in the final model. Multicollinearity tests were performed for the independent variables by evaluating variance inflation factors (VIF). The Hosmer-Lemeshow (HL) test was used to evaluate the fit of the logistic regression model. A residual analysis was performed using Cook’s distance to evaluate the fit of the binary logistic regression model to the data. The statistical analysis considered all the valid information and the loss of any information is indicated in the tables. All statistical analyses were performed using the Statistical Package for Social Sciences, v 22.0 (IBM SPSS Statistics for Windows, Armonk, USA).

## Results

In 2020, during the COVID-19 pandemic, 1,719 asynchronous dental teleconsulting sessions were conducted in Brazil. The highest demand was from the Southeast region (n = 732; 42.6%), followed by the Midwest (n = 647; 37.6%), Northeast (n = 190; 11.1%), South (n = 126; 7.3%), and North (n = 24; 1.4%).

Regarding dentist satisfaction, 73.3% were dissatisfied/neither satisfied nor satisfied. Concerning patient referral to other levels of care, 65.0% of the dentists who answered this question (77.1%) said teleconsulting avoided it. The majority of the questions were submitted by female dentists (59.7%), FHS specialist (55.3%), and general dental practitioners (37.6%). Most teleconsulting questions and answers were submitted during working hours (81.6% and 68.1%, respectively) and were related to semiology (33.2%), followed by clinical dentistry (32.8%) (Table 1).

**Table 1:** Descriptive analyses of the dental teleconsulting profile of the Telehealth Brazil Networks Program (2020) (n = 1,719).

Variable	Frequency n(%)
Satisfaction	
Dissatisfied/Neither satisfied nor dissatisfied	1260 (73.3)
Satisfied	459 (26.7)
Avoided referral*	
Yes	862 (65.0)**
No	464 (35.0)**
Sex*	
Female	1011 (59.7)**
Male	683 (40.3)**
Dentist specialty	
Family health strategy	950 (55.3)
General dental practitioner	646 (37.6)
Specialist	123 (7.2)
Time of question	
Within working hours	1402 (81.6)
Outside working hours	317 (18.4)
Time of answer	
Within working hours	1170 (68.1)
Outside working hours	549 (31.9)
Dental field	
Semiology	570 (33.2)
Clinical dentistry	564 (32.8)
Services	348 (20.2)
Oral medicine	122(7.1)
Health Promotion and Prevention	94 (5.5)
Pharmacology	21 (1.2)

*Variables with missing data. **Values for valid data. Sex (n = 1,694); Avoided referral (n = 1,326).

Only the covariates “avoided referral” and “dental field” were statistically associated with the PHC dentist satisfaction with teleconsulting in the unadjusted OR analysis. In the adjusted binary logistic regression analysis, the dentists reporting that teleconsulting avoided patient referral to other care levels were more likely to be satisfied with the teleconsultant’s answer (OR = 1.55, 95%CI: 1.17–2.04, p = 0.002). Dentists submitting oral medicine questions were significantly less satisfied with the teleconsultant feedback (OR = 0.61, 95%CI: 0.43–0.87, p = 0.006), compared with clinical dentistry (Table 2).

**Table 2:** Factors associated with dental teleconsulting satisfaction in the Telehealth Brazil Networks Program (2020) (n = 1,719).

Variable	Frequency of satisfaction (%)	Unadjusted OR (95%CI)	p-value	Adjusted OR (95%CI)	p-value
Sex*					
Male	26.5	0.99 (0.80–1.24)	0.961	–	-
Female	26.6	1			
Dental specialty					
Family Health Strategy	28.8	1.25 (1.00–1.57)	0.053		
Specialist	22.0	0.87 (0.55–1.38)	0.551	–	-
General dental practitioner	24.5	1			
Time of question					
Outside working hours	26.8	1.01 (0.77–1.33)	0.960	–	-
Within working hours	26.7	1			
Time of answer					
Outside working hours	26.8	1.01 (0.80–1.26)	0.962	–	-
Within working hours	26.7	1			
Avoided referral*					
Yes	28.1	1.58 (1.20–2.07)	0.001	1.55 (1.17–2.04)	0.002
No	19.8	1		1	
Dental field					
Semiology	31.4	1.21 (0.94–1.56)	0.148	1.22 (0.89–1.68)	0.213
Services	31.1	1.19 (0.78–1.83)	0.415	1.04 (0.64–1.71)	0.864
Oral medicine	18.7	0.61 (0.44–0.84)	0.003	0.61 (0.43–0.87)	0.006
Health promotion and prevention	19.1	0.63 (0.36–1.08)	0.092	0.65 (0.36–1.19)	0.164
Pharmacology	19.0	0.62 (0.21–1.87)	0.398	0.67 (0.19–2.38)	0.534
Dental clinic	27.5	1		1	

*Variables with missing data. Avoided referral n = 1,326; Sex n = 1,694; OR = odds ratio; CI = confidence interval

The statistical model had a good fit, with a HL p-value of 0.953, VIF values close to 1.0, suggesting the absence of collinearity, and Cook´s distance values lower than 1.0.

## Discussion

The main finding of this study raises a concern, since only a minor part of the dentists (26.7%) reported being satisfied with the service, in contrast to the results found in previous studies.^16-18^ Dentists answered the close-ended questions on the SMART platform according to their level of satisfaction with teleconsulting, but did not specify their criteria. Professional satisfaction with telehealth services^11-13^ should be addressed from several perspectives,^19^ such as the professional’s profile and demographics (sex and specialty),^11,13^ care-related contexts and experiences (type of practice and system, workflow, and amount of technical support available),^11,13^ and environmental and regional considerations.^11^ Poor quality of service equipment and information received^12,13^ affects the use of telehealth systems, has a negative impact on satisfaction, and discourages professionals from using the system.^12^

The distribution of teleconsulting in Brazil followed the same trend as in a previous study^18^. Female dentists submitted the highest number of teleconsulting questions, as observed previously.^17,20^ This may reflect the great adherence of women to the program^19^ or their prevalence in offering the service.^16,17^ However, sex was not significantly associated to satisfaction with teleconsulting sessions.

The major demand of teleconsulting by FHS dentists and generalists, and the lack of statistical association with satisfaction was expected based on the Brazilian PHC profile. In Brazil, the healthcare system guarantees the right to health care to all citizens. The PHC organizes and guides health demands, including oral health ^21^, and is the entry point for initial care, being responsible for solving most of the population’s problems.^21-23^ Health problems not solved in PHC are referred to specialized services^3,23^. Then, teledentistry helps solve the questions from PHC professionals,^1-5,23^ qualifies the patient referral process, and improves the cost/benefit ratio of the state’s health service.^3,23^

According to SMART, teleconsulting is the second most common service offered by telehealth centers, and asynchronous activities are used mainly by PHC professionals,^24^ probably because of the convenient schedule.^25^ Asynchronous teleconsulting can be scheduled at the professional’s convenience; however, teleconsulting services must favor and encourage access to the platform as part of the work routine.^20^ Although most teleconsulting sessions occurred during working hours, some were submitted in the evening, as observed elsewhere.^17^ Evening requests commonly result from work overload, such as low dentist-to-patient ratio and high demand for more complex treatments, because of the high incidence of oral diseases that require longer treatment^13^. Besides that, time connectivity issues^20^ or even infrastructure limitations^26^ are factors that could influence the professional satisfaction.^13^ Nevertheless, the timing of teleconsulting questions and answers was not statistically associated with the PHC dentist satisfaction.

Effectiveness and resolvability^3,11^ influences satisfaction, and are related to how well a health service can solve a problem based on its level of competence.^19^ Teleconsulting sessions between dentists can avoid unnecessary patient referrals and consequently improve the resolvability of PHC.^16,19^ Although there were some missing data for “patient referrals”, 65.0% of the dentists answering the question said that teleconsulting avoided these referrals, and were more likely to be satisfied with the teleconsultant’s answer. The feedback from professionals must be encouraged so that the program can be adequately evaluated.^17,19^ Telehealth has been reported to be effective in avoiding unnecessary referrals of patients to other care levels.^16^ Nevertheless, patient referral to another level of care does not necessarily mean that the teleconsulting did not solve the issue,^1^ considering that the data collected from the platform does not provide information on whether the referral was necessary or not.

The type of care and the “categories of care” packaged into the specialty fields influence one’s satisfaction with the service.^11^ Most of the questions related to semiology and clinical dentistry were expected, since they are constant themes associated with PHC – responsible for providing comprehensive care, including health promotion, diagnoses, treatments, and rehabilitation.^22^ Questions related to health promotion and prevention, pharmacology, and services were not statistically associated with PHC dentist satisfaction. However, dentists who submitted oral medicine questions were significantly less satisfied with the teleconsulting feedback, which is a cause for concern. Brazilian teleconsultants are usually experts in a particular field^3,5^, but may not be aware of the reality of PHC. In addition, they may suggest exams performed by a specialist to obtain an accurate diagnosis^22^. On the other hand, the high number of oral medicine questions regarding telehealth programs^16,17,23^ and the difficulties in diagnosing PHC-related oral diseases^3,16,17,20^ have also attracted attention. Oral health teams in Brazil have performed poorly in the early detection of oral cancer; moreover, in many cities there are no reference services for these cases.^26^ In addition, aspects related to the infrastructure and organization of the service^13^ may influence the ability of professionals to follow the guidance received in the teleconsulting, affecting their satisfaction.

During the COVID-19 pandemic, social distancing measures to prevent contamination resulted in elective dental procedures being suspended and only urgent care being maintained in Brazil.^27^ This affected PHC and dental specialty services,^27^ and resulted in a considerable drop in the average number of cancer diagnoses in the country with the start of the pandemic.^28^ This outcome reinforces the importance of teledentistry^28^ as a valuable tool for initial assessment and diagnosis of potentially malignant disorders and early-stage oral cancer,^29^ and also for telemonitoring.^2^ Therefore, PHC professionals must be very satisfied with the program and the program must work well, so that both the program and the professionals can better support the population.

Some studies have evaluated patient^30-33^ and professional^33^ satisfaction with the use of teledentistry during the COVID-19 pandemic. However, these studies are not comparable with the present study because they evaluated synchronous teleconsultations and not asynchronous teleconsulting. Synchronous teleconsultations allow a real-time conversation between the health professional and the patient^30-33^, while asynchronous teleconsulting consists of offline communication between two health professionals.^3,5^ In addition, this was the first investigation of the factors associated with the PHC dentists’ satisfaction with asynchronous dental teleconsulting of the Telehealth Brazil Networks Program and involved an important point in time (COVID-19) for evaluating remote healthcare support.^3,6,16^

Limitations of this study involve the use of a secondary database, since the reasons for dissatisfaction and the lack of dentists’ feedback could be investigated. The low level of satisfaction of PHC dentists with the program is of concern, as it may discourage the use of the tool or its recommendation to other professionals. One change that could be made to the program platform would be to give professionals the opportunity to explain the reasons for their dissatisfaction with the teleconsulting session. Future research delving into the reasons for the satisfaction/dissatisfaction of professionals should clarify these issues and gear actions toward improving program results. Also, new investigations after the initial period of the pandemic should assess its impact on these results.

## Conclusions

PHC dentists showed low satisfaction rates for the asynchronous dental teleconsulting of the Telehealth Brazil Networks Program in 2020. Professionals who gave patient referral feedback and who reported that teleconsulting avoided patient referral to other care levels were more likely to be satisfied with the teleconsultant’s answer. Dentists who submitted oral medicine questions were significantly less satisfied with the teleconsulting feedback. Feedback from PHC professionals must be encouraged to enable better evaluation of the program.
